# QuickStats

**Published:** 2013-04-26

**Authors:** Linda F. McCaig, Michael Albert

**Figure:**
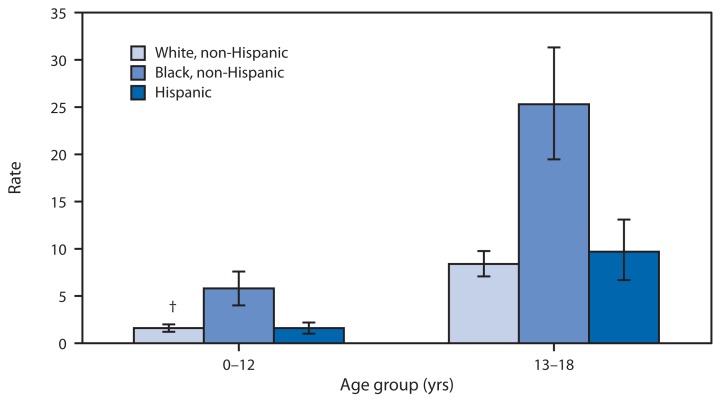
Average Annual Rate of Emergency Department Visits for Assault^*^ Among Persons Aged ≤18 Years, by Age Group and Race/Ethnicity — United States, 2005–2010 ^*^ Per 1,000 population, based on 6-year annual average. Assault was determined if any one of the following was the first-listed E-code: 960–969, homicide and injury purposely inflicted by other persons; 979, terrorism; 999.1, late effect of injury due to terrorism. ^†^ 95% confidence interval.

During 2005–2010, approximately 388,000 emergency department visits were made each year by persons aged ≤18 years who had been injured by assault, an overall rate of 5.0 visits per 1,000 persons per year. The visit rate for assault for non-Hispanic blacks aged 13–18 years was 25.3 per 1,000 population, which was higher than the 8.4 rate for non-Hispanic whites and the 9.7 rate for Hispanics. Among children aged 0–12 years, the visit rate also was higher among non-Hispanic blacks (5.7) than among non-Hispanic whites (1.6) or Hispanics (1.6).

**Source:** National Hospital Ambulatory Medical Care Survey 2005–2010. Available at http://www.cdc.gov/nchs/ahcd.htm.

